# P04.65. Development and validation of an instrument for measuring decision making about complementary and alternative medicine (CAM) use among cancer patients

**DOI:** 10.1186/1472-6882-12-S1-P335

**Published:** 2012-06-12

**Authors:** J Mao, K Desai, D Watkins-Brunner, E Frankel, S Palmer, S Xie, F Barg, K Armstrong

**Affiliations:** 1 Perelman School of Medicine at University of Pennsylvania, Philadelphia, USA

## Purpose

Despite cancer patients’ extensive use of complementary and alternative medicine (CAM), validated instruments to measure decision making factors related to CAM use are lacking. We sought to develop and validate an instrument, Decision Making in CAM (DMCAM), to measure individual cancer patients’ perceived benefit, barriers, and social norms related to CAM use.

## Methods

The 17-item instrument was developed using the Theory of Planned Behavior (TPB) as a theoretical framework. Literature review, qualitative interviews, expert content review, and cognitive interviews were used to develop the instrument which was then administered to 317 outpatient oncology patients. Reliability and validity of DMCAM scores were examined including factor structure, internal consistency, content, and construct validity.

## Results

The DMCAM had a 3-factor structure: expected benefits, perceived barriers, and subjective norms related to decision making about CAM use by cancer patients. These three domains had Eigen values of 4.79, 2.37, and 1.43, and together explained over 57.2% of the variance. The 4-item expected benefits, 7-item perceived barriers, and 4-item subjective norms domain scores each had an acceptable internal consistency (Cronbach’s alpha coefficient) of 0.91, 0.76, and 0.75 respectively. As expected, CAM users had higher expected benefits (65.2 vs. 52.1, p<0.001), lower perceived barriers (43.9 vs. 50.7, p<0.001), and more positive subjective norms (52.3 vs. 45.2, p<0.001) than those who did not use CAM.

## Conclusion

The DMCAM instrument produced reliable and valid scores that measured decision factors related to CAM use for cancer patients. Incorporation of the DMCAM in prospective research will help to determine how these factors may affect CAM use during cancer treatment and survivorship. Further, the use of this instrument in racially/ethnically diverse groups may help explain the variations in CAM use by cancer patients in specific populations.

